# Prenatal antidepressant exposure is associated with *in utero* changes in cortical thickness in the fetal brain

**DOI:** 10.3389/fnins.2026.1830026

**Published:** 2026-08-12

**Authors:** Kevin M. Cook, Jung-Hoon Kim, Nickie Andescavage, Kushal Kapse, Catherine Limperopoulos, Josepheen De Asis-Cruz

**Affiliations:** Developing Brain Institute, Children’s National Hospital, Washington, DC, United States

**Keywords:** antidepressant, brain, cortical thickness, fetal, limbic, maternal depression, SSRI

## Abstract

**Introduction:**

Approximately 1 in 8 perinatal women have major mood disorders, most commonly major depressive disorder, which is often comorbid with anxiety disorders. Despite well-established risks of untreated mood disorders, many women choose to forgo antidepressant treatment due to concern about effects on the developing fetus, despite unclear evidence of its impact. We examined the association between prenatal maternal antidepressant use and fetal brain cortical thickness.

**Methods:**

Retrospective analyses of a cohort of prospectively recruited maternal-fetal pairs that underwent fetal MRI were used to assess the impact of antidepressant exposure. Cortical thickness was measured for 96 structural fetal MRI scans, 48 with and 48 without antidepressant exposure across 82 brain regions at mean gestational ages of 31.0 ± 4.2 and 30.9 ± 4.9 weeks. Using linear mixed effect models, controlling for gestational age at scan, global cortical thickness, and repeat scans, we tested differences between antidepressant exposed and unexposed fetuses. Subsequently, models were repeated with the inclusion of maternal stress to assess its impact on the associations.

**Results:**

Following multiple comparison correction, 11 regions exhibited significant differences between the two groups. Exposed fetuses showed greater cortical thickness in the bilateral superior orbital gyri (*p* = 0.001) and superior temporal poles (*p* = 0.002) as well as the right middle (*p* = 0.001) and inferior (*p* = 0.004) orbital gyri and right parahippocampal gyrus (*p* < 0.001). The unexposed infants showed greater cortical thickness in the left postcentral *(p* < 0.001), angular (*p* = 0.004), and inferior parietal (*p* = 0.003) gyri and the right middle cingulate gyrus (*p* = 0.003). Once maternal stress was accounted for, however, there was significant attenuation of the relationship between exposure and cortical thickness.

**Discussion:**

*In utero* exposure to antidepressant medications are associated with changes in cortical thickness in limbic, paralimbic, and sensorimotor regions. Our findings mirror prior work suggesting that antidepressant exposure is associated with increases in structural development of limbic and paralimbic regions observed in those exposed later in life. However, attenuation of findings stemming from the inclusion of maternal stress suggests a more complex relationship between maternal psychopathology, antidepressant use, and fetal development that requires further examination.

## 1 Introduction

In the general population, anxiety and mood disorders are among the most common neuropsychiatric conditions, with as much as 25% of women report actively experiencing depressive symptoms and 21% experiencing anxiety symptoms (Terlizzi and Zablotsky, 2024). While treatment rates for individuals in North America and Europe are relatively high compared to the rest of the world (Mekonen et al., 2021) the majority of women are not screened or treated (Vesga-López et al., 2008), despite availability of numerous therapeutic approaches such as psychotherapy and medication.

Within the general population, pregnancy is a period of elevated stress (Marr et al., 2023; Kingston et al., 2012) and is associated with significantly increased rates of mood and anxiety disorders which persist and affect over 10% of pregnancies (Dietz et al., 2007; Ashley et al., 2016; Van Niel and Payne, 2020). Concerns about psychiatric medication, however, are common given uncertainty about the vulnerability of the developing brain throughout pregnancy (McDonagh et al., 2014; Eakley and Lyndon, 2022). Despite recommendations from the American College of Obstetricians and Gynecologists supporting the initiation and continued use of psychiatric medication including antidepressants due to the adverse effects of psychiatric illness (Committee on Clinical Practice Guidelines—Obstetrics, 2023), uncertainty on the long-term impacts of their use remains. As many as 80% of patients taking medications for mood and anxiety disorders discontinue use by the end of pregnancy (Logue et al., 2023). While this high rate of discontinuation can be managed in some women through alternative, non-pharmacological treatment approaches or remain in remission, some patients will experience a reemergence or increased severity of symptoms controlled by their medication. Discontinuation poses a significant problem, however, because experience of maternal psychological distress has previously been linked to changes in structural and functional brain development in neonates and fetuses including reduced cortical maturation (Wu et al., 2020; De Asis-Cruz et al., 2020; Marr et al., 2023), as well as an elevated risk for neurodevelopmental delays and disorders later in childhood compared to unexposed children (Werchan et al., 2024; Mareckova et al., 2020; Lautarescu et al., 2022; Power et al., 2023). Therefore, the balance between mitigating the effects of a pregnant person’s psychiatric disorder and its treatment on neurodevelopment is a challenging task.

Potential concerns about antidepressant medication use in pregnancy may stem from studies comparing children and adolescents with prenatal exposure to antidepressant medication compared to their unexposed peers. Children with prenatal exposure are more likely to delay starting elementary school (Kragholm et al., 2018), have higher rates of internalizing and externalizing problems (Oberlander et al., 2007), and have an increased risk for neurodevelopmental disorders (Clements et al., 2015; Rai et al., 2013; Bhat et al., 2023). However, growing evidence suggests that associated confounding factors may in fact be driving these relationships rather than antidepressant exposure itself (Suarez et al., 2022; Payne, 2021). For example, studies have found that controlling for maternal depression during childhood explains the relationship between fetal exposure and childhood depression – although prenatal antidepressant exposure was still associated altered brain growth, including increased cortical thickness in the left superior parietal and lateral occipital cortex (Moreau et al., 2023). Exposure has also associated with reduced cerebral gray matter, which is particularly pronounced in the amygdala and fusiform gyrus at 7 years of age; however, these differences wane by adolescence (Koc et al., 2023). More recent work in neonates and infants have potential to curtail the numerous postpartum and early life confounds by comparing brain development more proximally to birth. Neonates exposed to antidepressants prenatally have exhibited significant changes in brain development (for review see Campbell and Oberlander, 2025), which include differences in brain volumes as well as structural and functional connectivity. Infants with prenatal antidepressant exposure was associated with gross changes in white matter structure (Campbell et al., 2021; Jha et al., 2016; Lugo-Candelas et al., 2018), including structures like the amygdala. The also exhibit changes in functional connectivity, particularly in sensory regions including increased auditory network connectivity (Rotem-Kohavi et al., 2019b), thalamic-motor connectivity (Salzwedel et al., 2016), and greater hubness in Heschl’s gyrus (Rotem-Kohavi et al., 2019a) compared to unexposed infants.

Although there is growing literature on the impact of antidepressants in early life, there is still a significant gap in our understanding of prenatal impacts of exposure. While there is a significant absence of literature on medication exposure specifically, maternal depression, anxiety, and stress have been associated with decreased brain volumes prenatally (Lu et al., 2022) and cortical complexity which were subsequently associated with neurodevelopmental concerns at 18-months (Wu et al., 2022). Finally, maternal distress may be associated with cortical thickness changes throughout the fetal period (De Asis-Cruz et al., 2023). This mirrors work in adults with depression which suggests that experience of Major Depressive Disorder is associated with changes in cortical structure and thickness (Colloby et al., 2011; Truong et al., 2013), and in particular that antidepressant use in adults with depression is associated with cortical thinning (Schmaal et al., 2017; Serrarens et al., 2025). Although the exact mechanism is unclear, there are proposed links between the observed thinning and neurotrophic signaling, chronic stress, and inflammation (Castrén and Monteggia, 2021; Poletti et al., 2019; Li et al., 2025). Given the convergence on cortical thickness differences in adults with depression and antidepressant use as well as fetal cortical development and maternal stress, there is an important question about the intersection of maternal depression, antidepressant use, and fetal cortical brain development.

The growing understanding of the impact of maternal mood and anxiety disorders on early brain development and subsequent neurobehavioral correlates is becoming increasingly clear, highlighting the perils of not treating expecting mothers. However, our understanding of the impacts of antidepressant medications on the developing fetus have been largely unexplored. Here we examined the association between antidepressant exposure and brain structure in late second and third trimesters of fetal development, allowing us to directly assess the association without the confounding impacts of postnatal experiences such as those related to parenting or as well as environmental influences which are often more attenuated in the intrauterine environment. Using cortical thickness which less confounded by brain size compared to volumetric approaches (Schwarz et al., 2016), we assessed the association of brain development between fetuses exposed and unexposed fetuses to establish that neurodevelopmental changes observed postnatally begin to emerge during prenatal life.

## 2 Materials and methods

### 2.1 Participants

Across multiple prospectively recruited cohort studies of pregnancy complications and their impacts on fetal brain development, we pooled healthy fetal cohorts without congenital conditions to characterize the relationship between cortical brain development and antidepressant exposure. Fetuses were between 18 and 40 weeks of gestation and had normal fetal ultrasounds and echocardiograms. Fetuses were excluded if they had known or suspected genetic or chromosomal abnormalities as were fetuses of pregnant women with complicating medical conditions (metabolic or genetic disorders, or with complications such as preeclampsia or gestational diabetes) or MRI contraindications. The institutional review board of Children’s National Hospital in Washington, DC approved these studies, and all research was performed in accordance with guidelines and regulations. Written informed consent was obtained from each pregnant person. Following scanning and a review from a fetal neuroradiologist, fetuses with significant pathology (e.g., brain injury) were excluded from the final analysis. From across these prospectively recruited healthy cohort studies, we conducted a retrospective review to identify fetuses where antidepressant use was reported at any point during pregnancy, based on parental reports. Given the retrospective nature of these analyses, specific information on dosage, time of use, and diagnoses were not available. While depression and anxiety measures were not collected across each of these cohorts, we used scans only from collections that contained Perceived Stress Scale (PSS) which was recorded in a plurality of collections (Cohen et al., 1983) as a poxy to measure overall maternal stress and its potential interaction with fetal cortical brain development. Only those with no or minimal stress were used in the unexposed group. Those with antidepressant exposure were then matched on a scan-by-scan basis as described below to their nearest unexposed match.

### 2.2 MRI acquisition and preprocessing

Fetal images were collected at two timepoints during pregnancy using a 1.5T MRI scanner (GE Healthcare, Milwaukee, WI), once during the second trimester and once in the third trimester. Sagittal, axial, and coronal anatomical T2 weighted images were collected using a single-shot fast spin echo sequence (SSFSE) with 1100 ms TR, 160 ms TE, 0.859 mm^3^ reconstructed voxel size, and 90° flip angle. Following a standard reconstruction approach (Kainz et al., 2015; Rueckert et al., 1999; Serag et al., 2012), tissue and regional anatomical segmentation was performed using coronal T2 images using an automated algorithm (Makropoulos et al., 2014; Zhao et al., 2022) which was subsequently inspected and corrected as needed by a trained researcher (KK). Cortical thickness was then calculated within each of the 82 anatomical regions from a fetal anatomical atlas (Gholipour et al., 2014) using a minimum line integral approach (Aganj et al., 2009) in MATLAB 2021b and averaged within each anatomical ROI to yield a mean cortical thickness per region as well as a global cortical thickness for the entire cerebrum.

### 2.3 Experimental design and statistical analyses

After identifying participants with antidepressant exposure, individual scan matching was performed to match exposed and unexposed fetuses on a scan-to-scan basis to account for an imbalance in unexposed fetuses and a difference in gestational age at scan between the two (exposed 30.7 ± 4.8 weeks, unexposed 32.6 ± 4.5 weeks). Each scan from an exposed fetus was matched to its nearest unexposed scan using MatchIt (Ho et al., 2011) v4.5.5 in R v4.5.0 to find pairs matching for gestational age at scan and sex. Following matching, we utilized linear mixed effect models in subsequent analyses to control for the repeated scans across some subjects, using subject ID as a random effect. This approach was used to account for intrasubject growth, development, and exposure factors within the model. Initially, a single linear mixed effects model was used to compare global cortical thickness between the two groups, using unexposed as the reference group, using GA at scan as fixed factors and subject ID as a random factor. Subsequently, similar linear mixed effects models were used to test differences in cortical thicknesses across all 82 ROIs using GA at scan, global cortical thickness as it varies between groups, and subject ID to account for the repeated scans. Multiple comparisons across ROIs were then controlled for using false discovery rate (FDR) correction (Benjamini and Hochberg, 1995) at *p* < 0.05 Models were then repeated with the inclusion of sex as a covariate of interest.

Subsequently, the same ROI-level models were repeated with maternal self-reported stress on the Perceived Stress Scale (PSS) included to assess the impact of maternal stress first within just the ROI showing the impact of exposure and then again across all ROIs. After the initial models were run, mediation analyses were performed with PSS as the mediator to assess for the explanatory impact of PSS in describing the exposure-thickness relationships across all 82 ROI.

## 3 Results

### 3.1 Subjects

A total of 207 MRI studies from 135 fetuses completed a fetal MRI visit. Of those a total of 48 scans from 42 fetuses had any in utero exposure to antidepressants (*n* = 6 with two scans). Following scan-wise matching, the resulting unexposed datasets comprised 48 scans from 34 fetuses (*n* = 14 with two scans) (Table 1 and Supplementary Figure 1). The two groups following matching were not different in gestational age at scan t(47) = 0.24, *p* = 0.811, nor were there differences in gestational age at birth, birth weight, gravida or para. Conversely, the matching models were unable to converge on a cohort that was matched on sex, resulting in the unexposed group having significantly more scans from male fetuses at 56% compared to 50% χ^2^ (1, *N* = 96) = 21.67, *p* < 0.001. The exposure group was also less divers with 73% of the cohort being white and also had significantly higher stress scores on the PSS t(47) = 7.53, *p* < 0.001. Of the exposed fetal scans, all but two had fetal exposure to Selective Serotonin Reuptake Inhibitor (SSRI) exposure, two had exposures to SSRIs and other medications, and two had exposures to multiple non-SSRI medications.

**TABLE 1:** Participant demographics.

Demographics		Unexposed	Exposed	*p*
Participants		34	42	
Scans		48	48	
Maternal characteristics				
Gravida		2 [1,4]	1 [1,5]	
Para		1 [0,3]	0 [0,1]	
Vaginal birth		34 (71%)	34 (71%)	0.999
Perceived Stress Scale (PSS)		7.08 (3.41)	14.85 (6.01)	<0.001*
Medication classes^†^	SSRI	–	46	
	NDRI	–	4	
	GABA analog	–	2	
	Phenyltriazine derivative (lamotrigine)	–	3	
Maternal race				0.005*
	American Indian/Alaskan Indian	0	0	
	Asian/Pacific Islander	2 (4.2%)	5 (10.4%)	
	Black/African American	12 (25.0%)	1 (2.1%)	
	Hispanic	5 (10.4%)	3 (6.3%)	
	Non-Hispanic White	29 (60.4%)	35 (72.9%)	
	Unknown	0 (0.0%)	4 (8.3%)	
Fetal characteristics				
GA at scan		31.03 (4.23)	30.85 (4.91)	0.811
Sex (male)		27 (56.25%)	24 (50.00%)	<0.001*
GA at birth		39.39 (1.79)	38.86 (1.73)	0.143
Birth weight (grams)		3178.8 (641.0)	3220.4 (474.4)	0.725

GA, gestational age; SSRI, Selective Serotonin Reuptake Inhibitor; NDRI, Norepinephrine-Dopamine Reuptake Inhibitor.

† Some subjects had multiple exposures, so the total number exceeds the sample size.

*Significant at *p* < 0.05.

### 3.2 Cortical thickness and exposure

**FIGURE 1:**
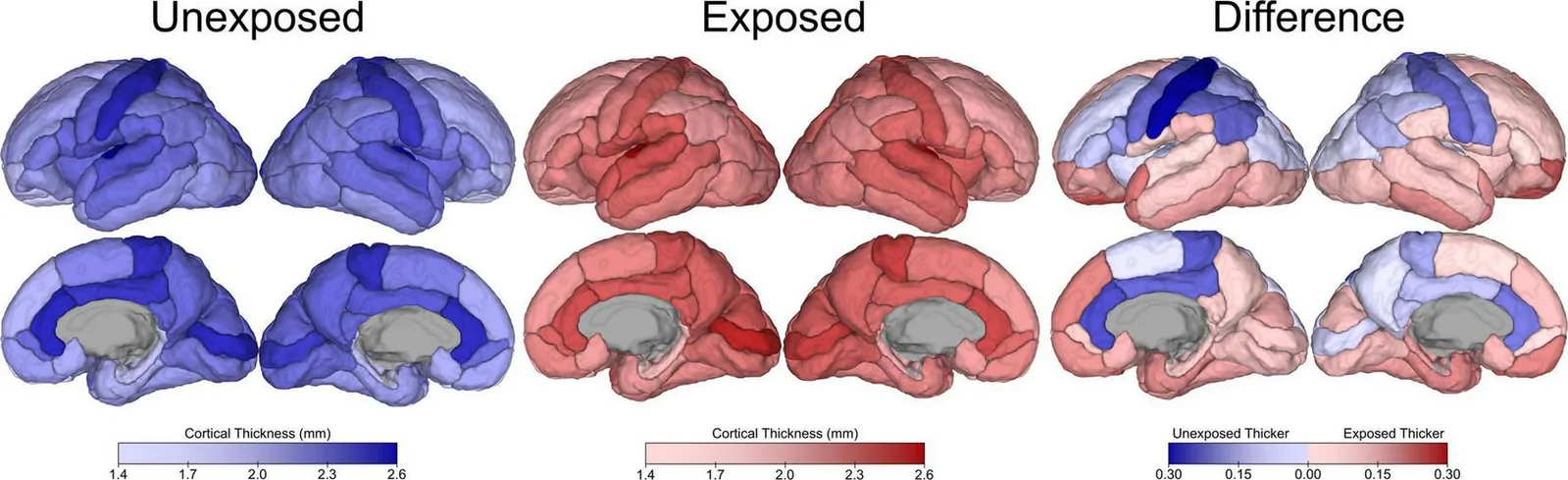
Cortical thickness by group. Mean cortical thickness for the unexposed (blue, left) and exposed groups (red, middle) across all regions. Difference between the average of both groups (right) for all regions with greater cortical thickness in unexposed in blue and regions with greater cortical thickness in the exposed group in red.

**FIGURE 2:**
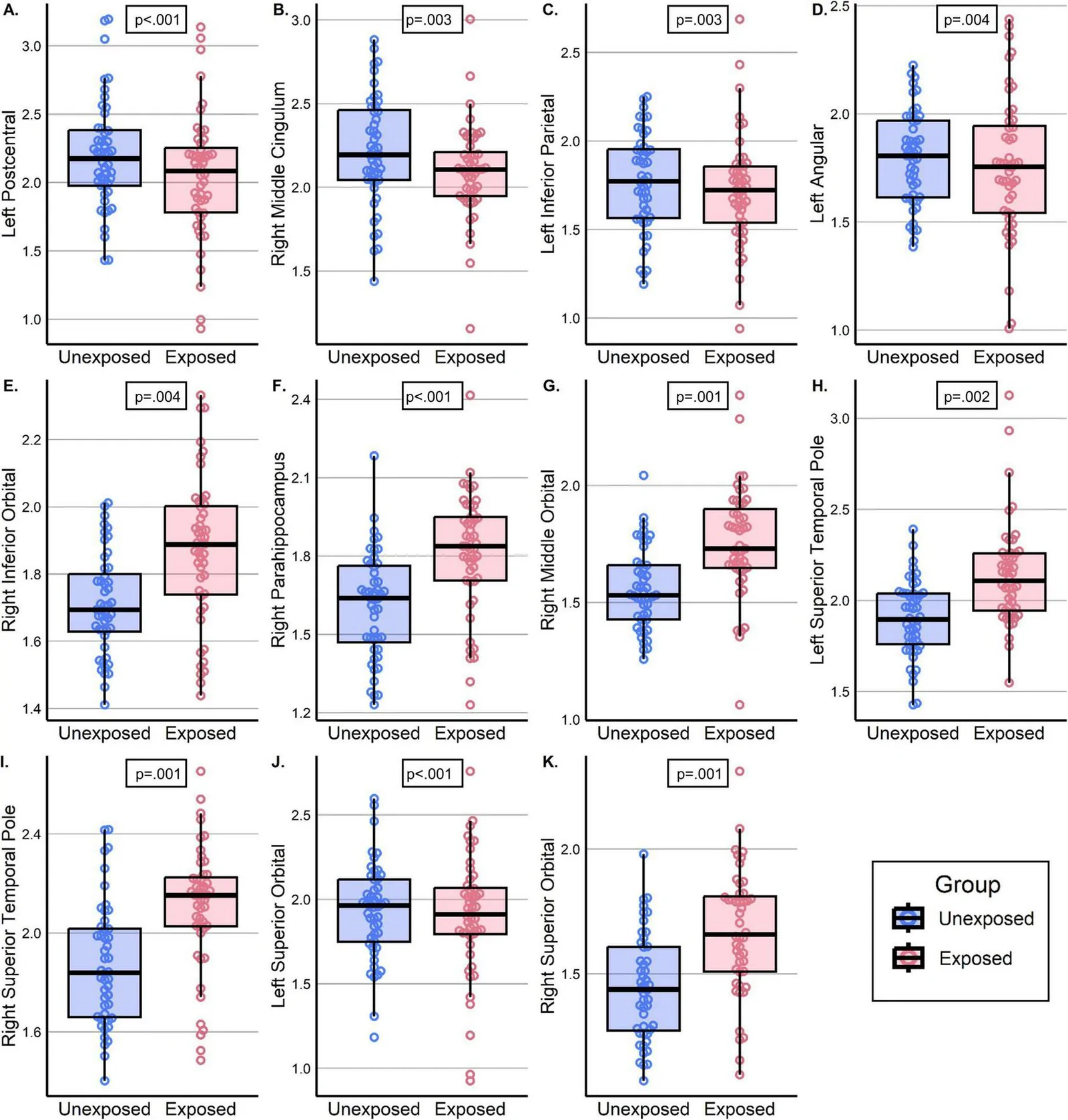
Differences in cortical thickness based on antidepressant exposure. Eleven regions exhibited differences in cortical thickness based on antidepressant exposure, with **(A–D)** reduced cortical thickness in exposed fetuses and **(E–K)** increased cortical thickness in exposed fetuses.

Overall, exposed fetuses exhibited increased global cortical thickness (mean 1.96 mm ± 0.18) compared to unexposed fetuses (mean 1.84 mm ± 0.16) *t*(47) = 3.33, *p* = 0.0020 (Figure 1). Regionally, cortical thickness also exhibited broad differences between exposed and unexposed fetuses. Linear mixed effects models yielded 11 ROIs (Figure 2 and Table 2) exhibiting significant cortical thickness differences between exposures after controlling for gestational age at scan, global cortical thickness, and repeated measures following FDR correction while an additional 4 were significant prior to FDR correction and remained trending following correction (see Supplementary Table 1 for complete results). Across 7 regions (bilateral superior orbital gyri and superior temporal poles, right middle and inferior orbital gyri, and right parahippocampal gyrus) antidepressant exposure was associated with increases in regional cortical thickness while exposure was associated with decreased cortical thickness in the left postcentral, angular, and inferior parietal gyri and the right middle cingulate gyrus. Importantly, although sex differed between the two groups, results did not change with the inclusion of sex in the model (Supplementary Table 2).

**TABLE 2:** Regions exhibiting significant cortical thickness differences.

ROI	Medication exposure		Gestational age		Global cortical thickness	
	β	*p*	β	*p*	β	*p*
Left postcentral gyrus	**−0.298**	<0.001*	**0.028**	0.003*	**1.202**	<0.001*
Right middle cingulate gyrus	**−0.197**	0.003*	0.005	0.455	**0.631**	0.004*
Left inferior parietal gyrus	**−0.175**	0.003*	**−0.023**	0.002*	**0.983**	<0.001*
Left angular gyrus	**−0.156**	0.004*	**−0.015**	0.017*	**1.008**	<0.001*
Left precentral gyrus	−0.139	0.010^†^	**0.023**	0.001*	**0.907**	<0.001*
Right precentral gyrus	−0.102	0.018^†^	0.006	0.267	**0.948**	<0.001*
Left parahippocamal gyrus	0.096	0.013^†^	0.006	0.14	**0.836**	<0.001*
Right inferior orbital gyrus	**0.118**	0.004*	0.005	0.315	**0.331**	0.013*
Right parahippocamal gyrus	**0.119**	<0.001*	**0.018**	<0.001*	**0.682**	<0.001*
Right middle orbital gyrus	**0.133**	0.001*	0.008	0.118	**0.519**	0.001*
Left superior temporal pole	**0.143**	0.002*	0.009	0.084	**0.792**	<0.001*
Right superior temporal pole	**0.158**	0.001*	**0.017**	0.003*	**0.603**	<0.001*
Right superior orbital gyrus	**0.170**	0.001*	−0.001	0.908	**0.405**	0.016*
Right rectus gyrus	0.171	0.014^†^	0.01	0.165	**0.654**	0.004*
Left superior orbital gyrus	**0.201**	<0.001*	0.006	0.354	**0.460**	0.013*

FDR adjusted *p*-values are presented, with those significant or trending denoted as follows: Bold and

*denote significant at *p* < 0.05 FDR corrected.

† Trending at *p* < 0.10 FDR corrected.

### 3.3 Maternal stress

Within the 11 ROI exhibiting significant association between cortical thickness and exposure, no ROI was significantly associated with PSS scores (*p* > 0.227 FDR corrected; Supplementary Table 3), although all ROI continued to exhibit a significant main effect for exposure except for the left angular, left inferior parietal, and right inferior orbital gyri (*p* > 0.392 FDR corrected). Repeating the same analyses utilizing all 82 ROI yielded no ROI exhibiting main effects for exposure or PSS following multiple comparison correction. However, the left superior orbital, right middle cingulate, right parahippocampal, and left postcentral gyri were all trending at *p* < 0.10 FDR corrected while PSS exhibited no significant or trending associations with regional cortical thicknesses with *p* < 0.508 FDR corrected (Supplementary Table 4). In the subsequent mediation analyses, PSS was not a significant mediator with ACME ranging from 0.014 to 0.020 (*p* > 0.480).

## 4 Discussion

Across a cohort of fetuses with varying exposure to antidepressant medications across pregnancy, we observed that exposure to medications such as SSRIs was associated with significant changes in cortical thickness during pregnancy compared to age-matched fetuses who were not exposed. In particular, exposed infants exhibited thicker cortices in limbic and paralimbic regions including medial temporal and inferior frontal regions. Conversely, sensorimotor regions were thinner in exposed infants, although only the left was significantly different following multiple comparison correction. Additionally, impacted regions remained largely consistent even after accounting for maternal stress.

Our findings suggest that some of the regions most strongly associated with changes in cortical development during the fetal period are the developing limbic and paralimbic structures in the brain, with exposure associated with increased cortical thickness. Limbic and paralimbic regions, including the orbitofrontal cortex, have a high density of serotonergic receptors, given that they are principal targets of dorsal raphe nucleus neurons (Roberts, 2011; Walker and Tadi, 2025). Although there is evidence that the placenta partially mediates the transfer of common antidepressants to the fetus, most medications ultimately do transfer from mothers to the fetuses (Zheng et al., 2022). With an excess of serotonin in particular, given the medications observed in this study, we may be observing a form of experience-dependent plasticity (Fu and Zuo, 2011) where this increase in synaptic serotonin is driving more activity and thus accelerated development of these brain regions. Typical maturation during the second half of pregnancy and throughout early infancy is marked by a dramatic global increase in cortical thickness throughout the brain (Xu et al., 2022; Wang et al., 2019; De Asis-Cruz et al., 2023). Given increases in thickness reflect maturation in typical development, one possible interpretation would be that the increase in thickness of limbic and paralimbic regions of exposed fetuses may suggest accelerated maturation of these regions, while the relative thinning of sensorimotor regions may suggest a delayed maturation – although longitudinal studies within fetuses are required to more directly test this hypothesis. In the absence of longitudinal work, it is also possible that the differences observed in the exposed fetuses are not truly maturation, but instead an interaction with more complex trajectories of cortical development which show variable development across different regions of the brain.

The limbic system is frequently identified as playing a role in the experience of depression and as a common target for treatment. Individuals with major depressive disorder often exhibited decreased volumes in limbic and paralimbic regions, including the orbitofrontal gyri and temporal poles (Mackin et al., 2025; Kronmüller et al., 2009; Grieve et al., 2013). Importantly, there are multiple studies which suggest that serotonergic medication alters structure and function of the limbic system. For example, while the hippocampi have been found to be significantly smaller in depression, there is evidence to suggest that prolonged SSRI use reduces the differences in volume compared to healthy controls (Boldrini et al., 2013), and that response to SSRI treatment in adults mitigates abnormal connectivity of the parahippocampal gyrus (Zhang et al., 2022). Moreover, boosting activity and connectivity of the orbitofrontal gyri and hippocampus via transcranial magnetic stimulation has been found to relieve depression symptoms (Han et al., 2023). It is possible that the increases in cortical thickness we observed mirror these differences in fetuses and may be associated with neurodevelopment concerns later in life – although long-term follow-ups are required before this can be assessed.

The other principal areas exhibiting associations with fetal antidepressant exposure are sensorimotor regions. Although there is significantly less literature in sensorimotor development and antidepressant exposure, there are notable developmental changes, particularly in neonates. Work with rodents has suggested that neonatal exposure to SSRI in rats was associated with less mature somatosensory development in early life (Lee, 2009), which is in keeping with our findings of reduced cortical thickness observed in our exposed fetuses. This work is mirrored in neonatal studies which have found that antidepressant exposure is associated with exposure being associated with reduced thalamic volumes (Jha et al., 2016). Exposed neonates also exhibit reduced functional connectivity between the thalamus and motor regions (Salzwedel et al., 2016) – particularly with the precentral gyri which have trending reductions in cortical thickness in our analyses. In neonates with antidepressant exposure, there is also significant differences in structural connectivity, particularly between postcentral gyri (Campbell et al., 2021), mirroring our findings which show significant left postcentral gyrus cortical thickness and right postcentral gyrus thickness, although the latter does not survive multiple comparison correction. Importantly, in each of these cases, there remained similar lingering questions about the association between the observed findings and maternal depression, anxiety, and stress which could not be fully assessed. Finally, untreated parental depression has been associated with worse motor development in their children (Liu et al., 2023), although worse motor development has also been observed in children exposed to antidepressants prenatally as well (Grove et al., 2018) although it is unclear if the latter is associated with residual depression symptoms or from medication exposure itself.

There is an important lingering question as to the impact of maternal symptoms both in SSRI exposed and unexposed fetuses. This is particularly true in the need to understand how unexposed fetuses of mothers who have clinically significant depressive and anxiety symptoms compare to either those with diagnoses and are exposed or those without either a diagnosis or exposure. While our study helps to elucidate differences with the inclusion of the PSS to account for maternal stress, prospective studies that more systematically assess maternal mood symptoms throughout pregnancy are required. A growing body of evidence demonstrates that maternal stress and depression are associated with significant changes in fetal and neonatal brain development even when symptoms are subclinical (Rotem-Kohavi et al., 2019b), although those findings did not replicate in our retrospective sample. Instead, our results suggest a more complicated picture. When not accounting for maternal stress, we observed cortical thickness differences. Inclusion of maternal stress using the PSS across only previously identified ROIs does not significantly change the findings, while examining the entire corpus of ROIs yields less clear. Only a third of previously significant ROIs maintain a trending relationship with exposure, while no ROIs exhibit even a borderline trending association with PSS scores. Similarly, mediation analyses suggest that PSS does not fully or partially mediate the associations between exposure and cortical thicknesses. As such, it is difficult to draw the conclusion that maternal distress explains the relationship between SSRI use and fetal brain development even though we observe higher stress in the mothers using SSRIs. This may be due to the lack of a direct measure of depression symptoms within our sample, but rather a reliance on perceived stress instead, which is a correlated but still distinct construct. As a result, we may be capturing a less precise proxy of depressive symptoms resulting in less clear findings than if we had a direct measure of depression available for these retrospective analyses. Similarly, stress may be playing a small but notable role in fetal brain development that, once accounted for in models, results in SSRI exposure effect sizes we are slightly underpowered to detect the trending but not significant findings. Although it should be noted that some evidence suggests that SSRI exposure may partially or fully mitigate the impact of depressive symptoms on brain connectivity (Rotem-Kohavi et al., 2019a), which may in tern generalize to brain structure.

There are some clear limitations to our current data that require additional research. First and most centrally, this work is retrospective analyses of otherwise health fetuses from prior data collections. As a result, there are constraints placed on our potential analyses and confounders given we are limited to data collected at their study visits which ultimately need to be considered when attempting to generalize our findings. Given limits on what was captured across these various studies we lack sufficient information on the dosage of medications, compliance, or their timings throughout pregnancy – instead we are limited to binary analyses of any versus no exposure. We are also unable to interrogate specific effects across different medications classes given our sample happened to be nearly entirely exposed to SSRI or in some cases SSRI and another medication. This means we are unable to assess whether the variable mechanisms of action of these medications are associated with different impacts fetal brain development. Although we did perform analyses utilizing only the cohort with SSRI exposure (Supplementary Table 5), there was not a meaningful change in the results outside of two regions (left precentral gyrus and right rectus) moving from trending to barely significant following FRD correction.

Second, although we observe differences between exposed and unexposed fetuses, the mechanistic drivers are unclear. Importantly, the two groups differ in both exposure but also in the fact that one cohort has a clinical diagnosis while the other does not. Additional work is required to recruit a group with psychiatric diagnoses who did not receive or discontinued medication during pregnancy to account for confounders related to mental health. Although we attempted to account for this with the inclusion of the PSS in secondary analyses, the PSS is not a direct analogue for clinical measures of depression or anxiety symptoms with studies suggesting imperfect overlap as they are measuring different constructs (Doty et al., 2022). Thus, we may not be fully capturing a more complex relationship between stress and both depression and anxiety symptoms. As such, residual confounding by maternal psychopathology may be contributing to the observed associations and that the antidepressant-related findings should be interpreted with appropriate caution Finally, work is ongoing to directly assess neurodevelopment later into childhood to test the long-term associations between exposures and fetal brain development, given evidence that later exposure including parental stress and household characteristics (Dhaliwal et al., 2020; Mezzacappa et al., 2017; Sujan et al., 2017) alongside socioeconomic status (Suarez et al., 2022) may better explain relationships previously attributed to the impact of antidepressant use in pregnancy.

Our study presents the first evaluation of the association between prenatal exposure to antidepressants and brain development in utero. As a result, we can directly assess the association with these medications without the confounding variables of parenting and other environmental exposures that are inherent in studying the impact of prenatal exposure later into childhood. Our findings, which mirror what is known about the impact of antidepressant exposure later in life but show the impact of exposures even prenatally. Although these results should be interpreted cautiously in light of potentially unaccounted for confounds stemming from maternal psychopathology.

## Data Availability

The raw data supporting the conclusions of this article will be made available by the authors, without undue reservation.
