# Supplementary material for: Prenatal antidepressant exposure is associated with in utero changes in cortical thickness in the fetal brain
Source: Front Neurosci. 2026 Aug 12;20:1830026. doi: 10.3389/fnins.2026.1830026 (PMC13506922; doi:10.3389/fnins.2026.1830026)
Supplement: Supplementary file 1 [file Data_Sheet_1.docx]

Supplementary Material

**Supplemental Figure 1: Distribution of GA at Scan and PSS Score**

Density plots of gestational age at scan for included scans plotted by group, and distribution of Perceived Stress Scale scores by group.

| Supplemental Table 1: Cortical Thickness Across all ROIs | | | | | | |
| --- | --- | --- | --- | --- | --- | --- |
|  | Exposure | | Gestational Age At Scan | | Global Cortical Thickness | |
| ROI | β | p | β | p | β | p |
| Precentral_L | *-0.139* | *.010^†^* | **0.023** | **.001*** | **0.907** | **<.001*** |
| Precentral_R | *-0.102* | *.018^†^* | 0.006 | .267 | **0.948** | **<.001*** |
| Frontal_Sup_L | -0.016 | .737 | *-0.013* | *.021^†^* | **1.062** | **<.001*** |
| Frontal_Sup_R | 0.021 | .570 | -0.009 | .049 | **0.995** | **<.001*** |
| Frontal_Sup_Orb_L | **0.201** | **<.001*** | 0.006 | .354 | **0.460** | **.013** |
| Frontal_Sup_Orb_R | **0.170** | **.001*** | -0.001 | .908 | **0.405** | **.016** |
| Frontal_Mid_L | -0.063 | .313 | -0.003 | .707 | **1.178** | **<.001*** |
| Frontal_Mid_R | -0.009 | .832 | -0.003 | .513 | **0.990** | **<.001*** |
| Frontal_Mid_Orb_L | 0.070 | .220 | **0.018** | **.018*** | **0.588** | **.004** |
| Frontal_Mid_Orb_R | **0.133** | **.001*** | 0.008 | .118 | **0.519** | **.001** |
| Frontal_Inf_Oper_L | -0.066 | .294 | 0.007 | .313 | **0.594** | **.006** |
| Frontal_Inf_Oper_R | -0.013 | .802 | -0.011 | .081 | **0.965** | **<.001*** |
| Frontal_Inf_Tri_L | -0.047 | .449 | -0.013 | .079 | **0.952** | **<.001*** |
| Frontal_Inf_Tri_R | -0.044 | .366 | -0.008 | .144 | **0.725** | **<.001*** |
| Frontal_Inf_Orb_L | 0.048 | .373 | 0.006 | .293 | **0.719** | **<.001*** |
| Frontal_Inf_Orb_R | **0.118** | **.004*** | 0.005 | .315 | **0.331** | **.013** |
| Rolandic_Oper_L | 0.003 | .954 | 0.001 | .841 | **1.249** | **<.001*** |
| Rolandic_Oper_R | 0.042 | .406 | -0.012 | .053 | **1.162** | **<.001*** |
| Supp_Motor_Area_L | -0.009 | .825 | 0.004 | .322 | **0.940** | **<.001*** |
| Supp_Motor_Area_R | -0.053 | .200 | 0.002 | .659 | **1.048** | **<.001*** |
| Olfactory_L | 0.073 | .243 | **0.024** | **.003*** | **0.793** | **.001** |
| Olfactory_R | 0.086 | .095 | **0.027** | **<.001*** | **0.690** | **<.001*** |
| Frontal_Sup_Medial_L | 0.074 | .099 | -0.009 | .068 | **1.062** | **<.001*** |
| Frontal_Sup_Medial_R | 0.074 | .192 | *-0.013* | *.044^†^* | **1.218** | **<.001*** |
| Frontal_Med_Orb_L | 0.019 | .787 | **0.019** | **.014*** | **0.816** | **.001** |
| Frontal_Med_Orb_R | 0.094 | .359 | **0.029** | **.012*** | **0.936** | **.006** |
| Rectus_L | 0.150 | .033 | **0.022** | **.002*** | **0.828** | **<.001*** |
| Rectus_R | *0.171* | *.014^†^* | 0.010 | .165 | **0.654** | **.004** |
| Insula_L | -0.028 | .544 | *0.013* | *.028^†^* | **1.147** | **<.001*** |
| Insula_R | 0.069 | .149 | **0.026** | **<.001*** | **0.802** | **<.001*** |
| Cingulum_Ant_L | -0.142 | .100 | **0.039** | **.001*** | **1.078** | **.001** |
| Cingulum_Ant_R | -0.160 | .065 | **0.025** | **.015*** | **0.928** | **.002** |
| Cingulum_Mid_L | -0.109 | .098 | 0.004 | .562 | **0.765** | **.001** |
| Cingulum_Mid_R | **-0.197** | **.003*** | 0.005 | .455 | **0.631** | **.004** |
| Cingulum_Post_L | -0.078 | .370 | -0.002 | .836 | **0.746** | **.007** |
| Cingulum_Post_R | -0.120 | .207 | **-0.033** | **.004*** | **1.313** | **<.001*** |
| Hippocampus_L | 0.042 | .376 | -0.006 | .163 | **0.382** | **.009** |
| Hippocampus_R | 0.019 | .686 | **-0.014** | **.010*** | *0.253* | *.077^†^* |
| ParaHippocampal_L | *0.096* | *.013^†^* | 0.006 | .140 | **0.836** | **<.001*** |
| ParaHippocampal_R | **0.119** | **<.001*** | **0.018** | **<.001*** | **0.682** | **<.001*** |
| Amygdala_L | 0.015 | .863 | -0.005 | .604 | **0.757** | **.009** |
| Amygdala_R | 0.100 | .260 | **-0.043** | **<.001*** | **1.231** | **<.001*** |
| Calcarine_L | 0.084 | .168 | **0.019** | **.014*** | **0.995** | **<.001*** |
| Calcarine_R | -0.028 | .684 | **0.041** | **<.001*** | **1.367** | **<.001*** |
| Cuneus_L | 0.086 | .058 | **-0.024** | **<.001*** | **1.224** | **<.001*** |
| Cuneus_R | 0.028 | .530 | -0.010 | .061 | **1.369** | **<.001*** |
| Lingual_L | 0.002 | .975 | 0.013 | .073 | **1.338** | **<.001*** |
| Lingual_R | 0.066 | .174 | **0.017** | **.005*** | **0.994** | **<.001*** |
| Occipital_Sup_L | -0.057 | .225 | -0.010 | .080 | **1.532** | **<.001*** |
| Occipital_Sup_R | 0.005 | .924 | -0.010 | .078 | **1.452** | **<.001*** |
| Occipital_Mid_L | -0.105 | .032 | -0.009 | .132 | **1.265** | **<.001*** |
| Occipital_Mid_R | -0.001 | .977 | -0.005 | .408 | **1.064** | **<.001*** |
| Occipital_Inf_L | -0.035 | .593 | **0.021** | **.013*** | **1.057** | **<.001*** |
| Occipital_Inf_R | 0.012 | .793 | 0.008 | .179 | **1.036** | **<.001*** |
| Fusiform_L | 0.044 | .243 | -0.008 | .086 | **1.132** | **<.001*** |
| Fusiform_R | 0.066 | .050 | -0.005 | .207 | **0.988** | **<.001*** |
| Postcentral_L | **-0.298** | **<.001*** | **0.028** | **.003*** | **1.202** | **<.001*** |
| Postcentral_R | -0.114 | .042 | **0.025** | **.001*** | **1.053** | **<.001*** |
| Parietal_Sup_L | -0.053 | .321 | -0.012 | .062 | **0.984** | **<.001*** |
| Parietal_Sup_R | 0.041 | .363 | -0.008 | .136 | **0.979** | **<.001*** |
| Parietal_Inf_L | **-0.175** | **.003*** | **-0.023** | **.002*** | **0.983** | **<.001*** |
| Parietal_Inf_R | -0.047 | .374 | **-0.028** | **<.001*** | **1.234** | **<.001*** |
| SupraMarginal_L | 0.015 | .835 | **-0.032** | **<.001*** | **1.007** | **<.001*** |
| SupraMarginal_R | -0.021 | .755 | **-0.036** | **<.001*** | **1.053** | **<.001*** |
| Angular_L | **-0.156** | **.004*** | **-0.015** | **.017*** | **1.008** | **<.001*** |
| Angular_R | -0.065 | .277 | -0.011 | .075 | **1.143** | **<.001*** |
| Precuneus_L | 0.007 | .832 | -0.007 | .074 | **0.909** | **<.001*** |
| Precuneus_R | 0.027 | .398 | *-0.008* | *.03*8*^†^* | **1.019** | **<.001*** |
| Paracentral_Lobule_L | -0.098 | .089 | **0.032** | **<.001*** | **1.112** | **<.001*** |
| Paracentral_Lobule_R | -0.067 | .278 | **0.042** | **<.001*** | **0.845** | **<.001*** |
| Heschl_L | -0.063 | .567 | **0.038** | **.003*** | **1.493** | **<.001*** |
| Heschl_R | -0.022 | .809 | **0.057** | **<.001*** | **1.010** | **.001** |
| Temporal_Sup_L | 0.031 | .482 | **-0.014** | **.014*** | **0.883** | **<.001*** |
| Temporal_Sup_R | 0.042 | .308 | -0.005 | .319 | **0.867** | **<.001*** |
| Temporal_Pole_Sup_L | **0.143** | **.002*** | 0.009 | .084 | **0.792** | **<.001*** |
| Temporal_Pole_Sup_R | **0.158** | **.001*** | **0.017** | **.003*** | **0.603** | **<.001*** |
| Temporal_Mid_L | -0.009 | .857 | -0.010 | .087 | **0.861** | **<.001*** |
| Temporal_Mid_R | 0.026 | .558 | **-0.016** | **.003*** | **0.675** | **<.001*** |
| Temporal_Pole_Mid_L | 0.110 | .068 | 0.011 | .156 | **0.845** | **<.001*** |
| Temporal_Pole_Mid_R | 0.156 | .021 | 0.011 | .047 | **0.824** | **<.001*** |
| Temporal_Inf_L | 0.005 | .916 | -0.005 | .351 | **1.008** | **<.001*** |
| Temporal_Inf_R | 0.064 | .136 | -0.004 | .409 | **0.685** | **<.001*** |
| Unadjusted p-values are presented with those significant or trending following correction as noted:  *****Significant at p<.05 FDR corrected  *^†^*Trending at p<.10 FDR corrected | | | | | | |

| Supplemental Table 2: Cortical Thickness Across all ROIs with Sex | | | | | | | | |
| --- | --- | --- | --- | --- | --- | --- | --- | --- |
|  | Exposure | | Gestational Age At Scan | | Sex | | Global Cortical Thickness | |
| ROI | β | p | β | p | β | p | β | p |
| Precentral_L | *-0.140* | *.010^†^* | **0.023** | **.001*** | -0.038 | .441 | **0.896** | **<.001*** |
| Precentral_R | -0.101 | .019 | 0.005 | .272 | 0.013 | .734 | **0.952** | **<.001*** |
| Frontal_Sup_L | -0.016 | .734 | *-0.013* | *.022^†^* | -0.009 | .844 | **1.060** | **<.001*** |
| Frontal_Sup_R | 0.022 | .556 | -0.009 | .049 | 0.029 | .408 | **1.003** | **<.001*** |
| Frontal_Sup_Orb_L | **0.202** | **<.001*** | 0.006 | .362 | 0.048 | .343 | **0.473** | **.011*** |
| Frontal_Sup_Orb_R | **0.171** | **.001*** | -0.001 | .899 | 0.038 | .413 | **0.415** | **.014*** |
| Frontal_Mid_L | -0.062 | .325 | -0.003 | .698 | 0.048 | .409 | **1.191** | **<.001*** |
| Frontal_Mid_R | -0.007 | .857 | -0.003 | .497 | 0.047 | .207 | **1.003** | **<.001*** |
| Frontal_Mid_Orb_L | 0.071 | .218 | **0.018** | **.018*** | 0.019 | .717 | **0.591** | **.004*** |
| Frontal_Mid_Orb_R | **0.134** | **.001*** | 0.007 | .124 | 0.037 | .316 | **0.528** | **<.001*** |
| Frontal_Inf_Oper_L | -0.064 | .309 | 0.007 | .321 | 0.081 | .167 | **0.616** | **.005** |
| Frontal_Inf_Oper_R | -0.011 | .832 | -0.011 | .078 | 0.057 | .242 | **0.981** | **<.001*** |
| Frontal_Inf_Tri_L | -0.043 | .480 | -0.013 | .080 | 0.109 | .058 | **0.975** | **<.001*** |
| Frontal_Inf_Tri_R | -0.042 | .383 | -0.009 | .137 | 0.060 | .178 | **0.742** | **<.001*** |
| Frontal_Inf_Orb_L | 0.049 | .350 | 0.006 | .315 | 0.085 | .086 | **0.746** | **<.001*** |
| Frontal_Inf_Orb_R | **0.118** | **.004*** | 0.005 | .319 | 0.007 | .844 | **0.333** | **.013*** |
| Rolandic_Oper_L | 0.003 | .947 | 0.001 | .846 | 0.016 | .735 | **1.253** | **<.001*** |
| Rolandic_Oper_R | 0.041 | .419 | -0.012 | .055 | -0.037 | .431 | **1.152** | **<.001*** |
| Supp_Motor_Area_L | -0.008 | .833 | 0.004 | .324 | 0.020 | .590 | **0.946** | **<.001*** |
| Supp_Motor_Area_R | -0.052 | .209 | 0.002 | .663 | 0.026 | .507 | **1.054** | **<.001*** |
| Olfactory_L | 0.072 | .248 | **0.024** | **.003*** | -0.037 | .517 | **0.782** | **.001*** |
| Olfactory_R | 0.086 | .095 | **0.027** | **<.001*** | 0.027 | .572 | **0.698** | **<.001*** |
| Frontal_Sup_Medial_L | 0.075 | .090 | -0.009 | .063 | 0.059 | .150 | **1.078** | **<.001*** |
| Frontal_Sup_Medial_R | 0.076 | .181 | *-0.013* | *.041^†^* | 0.059 | .263 | **1.235** | **<.001*** |
| Frontal_Med_Orb_L | 0.018 | .808 | **0.019** | **.014** | -0.038 | .576 | **0.807** | **.001*** |
| Frontal_Med_Orb_R | 0.091 | .372 | **0.030** | **.012** | -0.088 | .361 | **0.907** | **.007*** |
| Rectus_L | 0.148 | .035 | **0.022** | **.002** | -0.060 | .355 | **0.812** | **<.001*** |
| Rectus_R | *0.172* | *.015^†^* | 0.010 | .168 | 0.017 | .793 | **0.658** | **.004** |
| Insula_L | -0.030 | .517 | *0.013* | *.026^†^* | -0.050 | .249 | **1.133** | **<.001*** |
| Insula_R | 0.068 | .154 | **0.026** | **<.001*** | -0.016 | .716 | **0.797** | **<.001*** |
| Cingulum_Ant_L | -0.142 | .100 | **0.039** | **.001*** | -0.051 | .522 | **1.060** | **.001*** |
| Cingulum_Ant_R | -0.161 | .064 | **0.025** | **.015*** | -0.034 | .672 | **0.918** | **.002*** |
| Cingulum_Mid_L | -0.111 | .093 | 0.004 | .546 | -0.053 | .390 | **0.751** | **.001*** |
| Cingulum_Mid_R | **-0.197** | **.003*** | 0.005 | .454 | -0.018 | .773 | **0.626** | **.005*** |
| Cingulum_Post_L | -0.077 | .377 | -0.002 | .840 | 0.006 | .945 | **0.747** | **.007*** |
| Cingulum_Post_R | -0.121 | .205 | **-0.032** | **.005*** | -0.041 | .642 | **1.302** | **<.001*** |
| Hippocampus_L | 0.044 | .345 | -0.006 | .145 | 0.074 | .096 | **0.401** | **.006*** |
| Hippocampus_R | 0.018 | .692 | **-0.014** | **.011*** | -0.010 | .824 | *0.251* | *.082^†^* |
| ParaHippocampal_L | *0.099* | *.009^†^* | 0.006 | .150 | 0.066 | .060 | **0.854** | **<.001*** |
| ParaHippocampal_R | **0.118** | **<.001*** | **0.018** | **<.001*** | -0.035 | .232 | **0.670** | **<.001*** |
| Amygdala_L | 0.016 | .855 | -0.005 | .601 | 0.032 | .685 | **0.766** | **.009*** |
| Amygdala_R | 0.094 | .275 | **-0.042** | **<.001*** | -0.199 | .015 | **1.177** | **<.001*** |
| Calcarine_L | 0.084 | .170 | **0.019** | **.015*** | 0.003 | .957 | **0.996** | **<.001*** |
| Calcarine_R | -0.029 | .679 | **0.041** | **<.001*** | -0.021 | .745 | **1.362** | **<.001*** |
| Cuneus_L | 0.088 | .050 | **-0.024** | **<.001*** | 0.073 | .078 | **1.244** | **<.001*** |
| Cuneus_R | 0.030 | .510 | -0.010 | .059 | 0.046 | .271 | **1.381** | **<.001*** |
| Lingual_L | 0.002 | .966 | 0.013 | .075 | 0.022 | .683 | **1.344** | **<.001*** |
| Lingual_R | 0.066 | .172 | **0.017** | **.006*** | 0.019 | .673 | **0.999** | **<.001*** |
| Occipital_Sup_L | -0.056 | .234 | -0.010 | .079 | 0.031 | .479 | **1.541** | **<.001*** |
| Occipital_Sup_R | 0.004 | .941 | -0.010 | .082 | -0.037 | .447 | **1.442** | **<.001*** |
| Occipital_Mid_L | -0.106 | .033 | -0.009 | .135 | -0.013 | .770 | **1.261** | **<.001*** |
| Occipital_Mid_R | -0.003 | .942 | -0.005 | .418 | -0.072 | .109 | **1.045** | **<.001*** |
| Occipital_Inf_L | -0.035 | .598 | **0.021** | **.013*** | 0.012 | .844 | **1.060** | **<.001*** |
| Occipital_Inf_R | 0.010 | .828 | 0.008 | .184 | -0.091 | .038 | **1.017** | **<.001*** |
| Fusiform_L | 0.046 | .221 | -0.008 | .078 | 0.060 | .087 | **1.148** | **<.001*** |
| Fusiform_R | 0.065 | .053 | -0.005 | .212 | -0.028 | .367 | **0.980** | **<.001*** |
| Postcentral_L | -0.301 | **<.001*** | 0.028 | **.003*** | -0.073 | .278 | **1.184** | **<.001*** |
| Postcentral_R | -0.114 | .042 | 0.025 | **.001*** | -0.015 | .765 | **1.049** | **<.001*** |
| Parietal_Sup_L | -0.052 | .328 | -0.012 | .062 | 0.017 | .731 | **0.988** | **<.001*** |
| Parietal_Sup_R | 0.041 | .373 | -0.008 | .140 | -0.026 | .539 | **0.972** | **<.001*** |
| Parietal_Inf_L | **-0.174** | **.003*** | **-0.023** | **.002** | 0.042 | .433 | **0.994** | **<.001*** |
| Parietal_Inf_R | -0.047 | .382 | **-0.028** | **<.001*** | 0.024 | .633 | **1.239** | **<.001*** |
| SupraMarginal_L | 0.016 | .816 | **-0.032** | **<.001*** | 0.047 | .474 | **1.020** | **<.001*** |
| SupraMarginal_R | -0.021 | .757 | **-0.036** | **<.001*** | 0.002 | .976 | **1.054** | **<.001*** |
| Angular_L | **-0.155** | **.004*** | **-0.015** | **.017** | 0.008 | .867 | **1.010** | **<.001*** |
| Angular_R | -0.066 | .268 | -0.011 | .079 | -0.046 | .414 | **1.130** | **<.001*** |
| Precuneus_L | 0.007 | .836 | -0.007 | .076 | -0.004 | .886 | **0.908** | **<.001*** |
| Precuneus_R | 0.028 | .379 | *-0.008* | *.036^†^* | 0.036 | .225 | **1.029** | **<.001*** |
| Paracentral_Lobule_L | -0.098 | .091 | **0.032** | **<.001*** | -0.008 | .884 | **1.109** | **<.001*** |
| Paracentral_Lobule_R | -0.067 | .280 | **0.042** | **<.001*** | -0.010 | .865 | **0.841** | **<.001*** |
| Heschl_L | -0.067 | .542 | **0.038** | **.002*** | -0.140 | .175 | **1.454** | **<.001*** |
| Heschl_R | -0.023 | .802 | **0.058** | **<.001*** | -0.022 | .800 | **1.006** | **.001*** |
| Temporal_Sup_L | 0.032 | .478 | **-0.014** | **.015*** | 0.009 | .829 | **0.885** | **<.001*** |
| Temporal_Sup_R | 0.042 | .298 | -0.004 | .346 | -0.074 | .052 | **0.840** | **<.001*** |
| Temporal_Pole_Sup_L | **0.144** | **.002*** | 0.009 | .087 | 0.021 | .612 | **0.798** | **<.001*** |
| Temporal_Pole_Sup_R | **0.157** | **.001*** | **0.017** | **.002*** | -0.029 | .509 | **0.594** | **<.001*** |
| Temporal_Mid_L | -0.008 | .872 | -0.010 | .086 | 0.031 | .495 | **0.870** | **<.001*** |
| Temporal_Mid_R | 0.024 | .583 | -0.016 | .003 | -0.062 | .131 | **0.660** | **<.001*** |
| Temporal_Pole_Mid_L | 0.114 | .056 | 0.011 | .143 | 0.086 | .120 | **0.858** | **<.001*** |
| Temporal_Pole_Mid_R | 0.154 | .024 | *0.011* | *.045^†^* | -0.049 | .436 | **0.811** | **<.001*** |
| Temporal_Inf_L | 0.006 | .896 | -0.005 | .337 | 0.062 | .151 | **1.028** | **<.001*** |
| Temporal_Inf_R | 0.063 | .144 | -0.004 | .419 | -0.035 | .388 | **0.676** | **<.001*** |
| Sex reference group is *male*  Unadjusted p-values are presented with those significant or trending following correction as noted:  *****Significant at p<.05 FDR corrected  *^†^*Trending at p<.10 FDR corrected | | | | | | | | |

| Supplemental Table 3: Cortical Thickness Across Significant ROIs with PSS | | | | | | | | |
| --- | --- | --- | --- | --- | --- | --- | --- | --- |
|  | Exposure | | Gestational Age At Scan | | Global Cortical Thickness | | Perceived Stress Scale | |
| ROI | β | p | β | p | β | p | β | p |
| Frontal_Sup_Orb_L | **0.209** | **.002*** | 0.006 | .379 | **0.463** | **.014*** | -0.001 | .832 |
| Frontal_Sup_Orb_R | **0.153** | **.013*** | 0.000 | .970 | **0.398** | **.019*** | 0.002 | .623 |
| Frontal_Mid_Orb_R | **0.130** | **.009*** | 0.008 | .121 | **0.518** | **.001*** | 0.000 | .914 |
| Frontal_Inf_Orb_R | *0.084* | *.081^†^* | 0.006 | .238 | **0.316** | **.018*** | 0.005 | .216 |
| Cingulum_Mid_R | **-0.242** | **.003*** | 0.007 | .374 | **0.611** | **.006*** | 0.007 | .311 |
| ParaHippocampal_R | **0.117** | **.003*** | **0.018** | **<.001** | **0.680** | **<.001** | 0.000 | .913 |
| Postcentral_L | **-0.309** | **.001*** | **0.028** | **.004*** | **1.197** | **<.001** | 0.002 | .823 |
| Parietal_Inf_L | -0.114 | .101 | **-0.025** | **.002*** | **1.009** | **<.001** | -0.009 | .131 |
| Angular_L | -0.069 | .267 | **-0.017** | **.007*** | **1.045** | **<.001** | -0.013 | .021 |
| Temporal_Pole_Sup_L | **0.130** | **.018*** | 0.009 | .084 | **0.784** | **<.001** | 0.002 | .640 |
| Temporal_Pole_Sup_R | **0.126** | **.025*** | **0.018** | **.002*** | **0.592** | **<.001** | 0.005 | .309 |
| Unadjusted p-values are presented with those significant or trending following correction as noted:  *****Significant at p<.05 FDR corrected  *^†^*Trending at p<.10 FDR corrected | | | | | | | | |

| Supplemental Table 4: Cortical Thickness Across all ROIs with PSS | | | | | | | | |
| --- | --- | --- | --- | --- | --- | --- | --- | --- |
|  | Exposure | | Gestational Age At Scan | | Global Cortical Thickness | | Perceived Stress Scale | |
| ROI | β | p | β | p | β | p | β | p |
| Precentral_L | -0.152 | .020 | **0.024** | **.001*** | **0.901** | **<.001*** | 0.002 | .718 |
| Precentral_R | -0.110 | .035 | 0.006 | .257 | **0.945** | **<.001*** | 0.001 | .765 |
| Frontal_Sup_L | -0.036 | .526 | *-0.013* | *.028^†^* | **1.053** | **<.001*** | 0.003 | .533 |
| Frontal_Sup_R | 0.002 | .965 | -0.008 | .066 | **0.987** | **<.001*** | 0.003 | .463 |
| Frontal_Sup_Orb_L | *0.209* | *.002^†^* | 0.006 | .379 | **0.463** | **.014*** | -0.001 | .832 |
| Frontal_Sup_Orb_R | 0.153 | .013 | 0.000 | .970 | **0.398** | **.019*** | 0.002 | .623 |
| Frontal_Mid_L | -0.078 | .304 | -0.002 | .751 | **1.171** | **<.001*** | 0.002 | .727 |
| Frontal_Mid_R | -0.001 | .983 | -0.003 | .492 | **0.993** | **<.001*** | -0.001 | .787 |
| Frontal_Mid_Orb_L | 0.006 | .929 | **0.020** | **.011*** | **0.557** | **.006*** | 0.009 | .112 |
| Frontal_Mid_Orb_R | 0.130 | .009 | 0.008 | .121 | **0.518** | **.001*** | 0.000 | .914 |
| Frontal_Inf_Oper_L | -0.006 | .939 | 0.006 | .426 | **0.620** | **.005*** | -0.009 | .175 |
| Frontal_Inf_Oper_R | 0.018 | .780 | -0.011 | .066 | **0.979** | **<.001*** | -0.005 | .388 |
| Frontal_Inf_Tri_L | 0.004 | .959 | -0.014 | .061 | **0.971** | **<.001*** | -0.007 | .244 |
| Frontal_Inf_Tri_R | -0.077 | .188 | -0.008 | .193 | **0.711** | **<.001*** | 0.005 | .321 |
| Frontal_Inf_Orb_L | 0.050 | .437 | 0.006 | .305 | **0.720** | **<.001*** | 0.000 | .949 |
| Frontal_Inf_Orb_R | 0.084 | .081 | 0.006 | .238 | **0.316** | **.018*** | 0.005 | .216 |
| Rolandic_Oper_L | 0.025 | .680 | 0.001 | .920 | **1.259** | **<.001*** | -0.003 | .525 |
| Rolandic_Oper_R | -0.019 | .750 | -0.010 | .090 | **1.136** | **<.001*** | 0.009 | .090 |
| Supp_Motor_Area_L | -0.021 | .645 | 0.004 | .305 | **0.934** | **<.001*** | 0.002 | .608 |
| Supp_Motor_Area_R | -0.073 | .143 | 0.002 | .613 | **1.044** | **<.001*** | 0.003 | .488 |
| Olfactory_L | 0.031 | .679 | **0.025** | **.002*** | **0.775** | **.001*** | 0.006 | .345 |
| Olfactory_R | 0.030 | .624 | **0.029** | **<.001*** | **0.667** | **<.001*** | 0.008 | .126 |
| Frontal_Sup_Medial_L | 0.070 | .190 | -0.009 | .075 | **1.061** | **<.001*** | 0.000 | .919 |
| Frontal_Sup_Medial_R | -0.010 | .883 | -0.011 | .084 | **1.183** | **<.001*** | 0.012 | .034 |
| Frontal_Med_Orb_L | 0.044 | .613 | **0.018** | **.018*** | **0.824** | **.001*** | -0.003 | .618 |
| Frontal_Med_Orb_R | 0.057 | .644 | **0.030** | **.012*** | **0.924** | **.007*** | 0.005 | .591 |
| Rectus_L | 0.131 | .112 | **0.022** | **.002*** | **0.821** | **<.001*** | 0.003 | .663 |
| Rectus_R | 0.114 | .164 | 0.011 | .122 | **0.631** | **.005*** | 0.008 | .211 |
| Insula_L | -0.073 | .199 | **0.014** | **.019*** | **1.128** | **<.001*** | 0.006 | .183 |
| Insula_R | -0.001 | .989 | **0.028** | **<.001*** | **0.773** | **<.001*** | 0.010 | .035 |
| Cingulum_Ant_L | -0.271 | .009 | **0.042** | **<.001*** | **1.028** | **.001*** | 0.018 | .037 |
| Cingulum_Ant_R | -0.266 | .010 | **0.027** | **.008*** | **0.882** | **.003*** | 0.015 | .076 |
| Cingulum_Mid_L | -0.162 | .041 | 0.005 | .458 | **0.741** | **.001*** | 0.008 | .224 |
| Cingulum_Mid_R | *-0.242* | *.003^†^* | 0.007 | .374 | **0.611** | **.006*** | 0.007 | .311 |
| Cingulum_Post_L | -0.117 | .257 | -0.001 | .907 | **0.734** | **.008*** | 0.006 | .492 |
| Cingulum_Post_R | -0.173 | .131 | **-0.031** | **.007*** | **1.292** | **<.001*** | 0.008 | .415 |
| Hippocampus_L | 0.051 | .359 | -0.006 | .157 | **0.385** | **.009*** | -0.001 | .751 |
| Hippocampus_R | 0.048 | .389 | **-0.015** | **.009*** | *0.264* | *.068^†^* | -0.004 | .351 |
| ParaHippocampal_L | 0.058 | .203 | 0.007 | .098 | **0.819** | **<.001*** | 0.006 | .134 |
| ParaHippocampal_R | *0.117* | *.003^†^* | **0.018** | **<.001*** | **0.680** | **<.001*** | 0.000 | .913 |
| Amygdala_L | -0.035 | .737 | -0.004 | .700 | **0.736** | **.012*** | 0.007 | .408 |
| Amygdala_R | 0.073 | .498 | **-0.042** | **.001*** | **1.219** | **<.001*** | 0.004 | .664 |
| Calcarine_L | 0.142 | .057 | *0.017* | *.024^†^* | **1.020** | **<.001*** | -0.008 | .185 |
| Calcarine_R | 0.000 | .996 | **0.040** | **<.001*** | **1.380** | **<.001*** | -0.004 | .555 |
| Cuneus_L | 0.061 | .265 | **-0.023** | **<.001*** | **1.213** | **<.001*** | 0.004 | .424 |
| Cuneus_R | -0.033 | .533 | -0.009 | .108 | **1.343** | **<.001*** | 0.009 | .057 |
| Lingual_L | 0.047 | .508 | 0.011 | .108 | **1.363** | **<.001*** | -0.007 | .264 |
| Lingual_R | 0.068 | .247 | 0.017 | .007 | **0.995** | **<.001*** | 0.000 | .943 |
| Occipital_Sup_L | -0.051 | .376 | -0.010 | .081 | **1.535** | **<.001*** | -0.001 | .844 |
| Occipital_Sup_R | -0.050 | .415 | -0.009 | .123 | **1.431** | **<.001*** | 0.008 | .120 |
| Occipital_Mid_L | -0.030 | .596 | -0.011 | .068 | **1.297** | **<.001*** | -0.011 | .033 |
| Occipital_Mid_R | 0.000 | .999 | -0.005 | .413 | **1.065** | **<.001*** | 0.000 | .968 |
| Occipital_Inf_L | 0.050 | .528 | *0.018* | *.024^†^* | **1.093** | **<.001*** | -0.012 | .072 |
| Occipital_Inf_R | -0.021 | .712 | 0.009 | .142 | **1.021** | **<.001*** | 0.005 | .314 |
| Fusiform_L | 0.047 | .306 | -0.008 | .089 | **1.133** | **<.001*** | 0.000 | .909 |
| Fusiform_R | 0.042 | .304 | -0.004 | .274 | **0.977** | **<.001*** | 0.003 | .297 |
| Postcentral_L | *-0.309* | *.001^†^* | **0.028** | **.004*** | **1.197** | **<.001*** | 0.002 | .823 |
| Postcentral_R | -0.078 | .243 | **0.024** | **.001*** | **1.068** | **<.001*** | -0.005 | .343 |
| Parietal_Sup_L | -0.043 | .506 | -0.012 | .062 | **0.988** | **<.001*** | -0.001 | .792 |
| Parietal_Sup_R | 0.035 | .524 | -0.008 | .151 | **0.977** | **<.001*** | 0.001 | .847 |
| Parietal_Inf_L | -0.114 | .101 | **-0.025** | **.002*** | **1.009** | **<.001*** | -0.009 | .131 |
| Parietal_Inf_R | -0.006 | .921 | **-0.029** | **<.001*** | **1.252** | **<.001*** | -0.006 | .276 |
| SupraMarginal_L | 0.130 | .111 | **-0.035** | **<.001*** | **1.064** | **<.001*** | -0.017 | .016 |
| SupraMarginal_R | 0.034 | .673 | **-0.037** | **<.001*** | **1.077** | **<.001*** | -0.008 | .243 |
| Angular_L | -0.069 | .267 | **-0.017** | **.007*** | **1.045** | **<.001*** | -0.013 | .021 |
| Angular_R | -0.002 | .974 | -0.013 | .048 | **1.168** | **<.001*** | -0.009 | .119 |
| Precuneus_L | 0.014 | .724 | -0.007 | .073 | **0.912** | **<.001*** | -0.001 | .754 |
| Precuneus_R | 0.013 | .737 | -0.008 | .050 | **1.013** | **<.001*** | 0.002 | .531 |
| Paracentral_Lobule_L | -0.144 | .039 | **0.033** | **<.001*** | **1.091** | **<.001*** | 0.007 | .242 |
| Paracentral_Lobule_R | -0.090 | .228 | **0.042** | **<.001*** | **0.835** | **<.001*** | 0.003 | .581 |
| Heschl_L | -0.091 | .487 | **0.038** | **.003*** | **1.483** | **<.001*** | 0.004 | .698 |
| Heschl_R | -0.036 | .735 | **0.058** | **<.001*** | **1.000** | **.001*** | 0.002 | .792 |
| Temporal_Sup_L | 0.049 | .355 | **-0.014** | **.013*** | **0.891** | **<.001*** | -0.003 | .560 |
| Temporal_Sup_R | 0.074 | .139 | -0.005 | .249 | **0.882** | **<.001*** | -0.005 | .261 |
| Temporal_Pole_Sup_L | 0.130 | .018 | 0.009 | .084 | **0.784** | **<.001*** | 0.002 | .640 |
| Temporal_Pole_Sup_R | 0.126 | .025 | **0.018** | **.002*** | **0.592** | **<.001*** | 0.005 | .309 |
| Temporal_Mid_L | 0.008 | .888 | -0.011 | .080 | **0.868** | **<.001*** | -0.002 | .620 |
| Temporal_Mid_R | 0.034 | .532 | **-0.017** | **.004*** | **0.678** | **<.001*** | -0.001 | .802 |
| Temporal_Pole_Mid_L | 0.041 | .566 | 0.012 | .107 | **0.807** | **<.001*** | 0.010 | .109 |
| Temporal_Pole_Mid_R | 0.176 | .028 | 0.011 | .052 | **0.825** | **<.001*** | -0.003 | .634 |
| Temporal_Inf_L | 0.020 | .727 | -0.005 | .325 | **1.013** | **<.001*** | -0.002 | .651 |
| Temporal_Inf_R | 0.048 | .359 | -0.004 | .467 | **0.678** | **<.001*** | 0.002 | .574 |
| Unadjusted p-values are presented with those significant or trending following correction as noted:  *****Significant at p<.05 FDR corrected  *^†^*Trending at p<.10 FDR corrected | | | | | | | | |

| Supplemental Table 5: Cortical Thickness Across all ROIs with Only SSRI Exposed Fetuses | | | | | | |
| --- | --- | --- | --- | --- | --- | --- |
|  | Exposure | | Gestational Age At Scan | | Global Cortical Thickness | |
| ROI | β | p | β | p | β | p |
| Precentral_L | **-0.148** | **.006*** | **0.022** | **.001*** | **0.916** | **<.001*** |
| Precentral_R | -0.098 | .026 | 0.006 | .263 | **0.947** | **<.001*** |
| Frontal_Sup_L | -0.014 | .766 | **-0.014** | **.020*** | **1.065** | **<.001*** |
| Frontal_Sup_R | 0.015 | .680 | *-0.010* | *.033^†^* | **1.004** | **<.001*** |
| Frontal_Sup_Orb_L | **0.189** | **.001*** | 0.004 | .483 | **0.475** | **.009*** |
| Frontal_Sup_Orb_R | **0.184** | **<.001*** | 0.000 | .954 | **0.395** | **.017*** |
| Frontal_Mid_L | -0.076 | .229 | -0.004 | .603 | **1.189** | **<.001*** |
| Frontal_Mid_R | -0.015 | .710 | -0.003 | .443 | **0.995** | **<.001*** |
| Frontal_Mid_Orb_L | 0.078 | .179 | **0.019** | **.016*** | **0.582** | **.005*** |
| Frontal_Mid_Orb_R | **0.126** | **.003*** | 0.007 | .121 | **0.520** | **.001*** |
| Frontal_Inf_Oper_L | -0.076 | .233 | 0.007 | .379 | **0.604** | **.006*** |
| Frontal_Inf_Oper_R | -0.017 | .754 | -0.011 | .074 | **0.970** | **<.001*** |
| Frontal_Inf_Tri_L | -0.049 | .434 | -0.013 | .083 | **0.951** | **<.001*** |
| Frontal_Inf_Tri_R | -0.041 | .401 | -0.008 | .163 | **0.722** | **<.001*** |
| Frontal_Inf_Orb_L | 0.052 | .345 | 0.007 | .256 | **0.713** | **<.001*** |
| Frontal_Inf_Orb_R | **0.128** | **.001*** | 0.006 | .198 | **0.319** | **.014*** |
| Rolandic_Oper_L | -0.003 | .957 | 0.001 | .894 | **1.253** | **<.001*** |
| Rolandic_Oper_R | 0.048 | .351 | -0.012 | .060 | **1.160** | **<.001*** |
| Supp_Motor_Area_L | -0.009 | .821 | 0.004 | .349 | **0.942** | **<.001*** |
| Supp_Motor_Area_R | -0.051 | .219 | 0.001 | .752 | **1.054** | **<.001*** |
| Olfactory_L | 0.076 | .232 | **0.024** | **.003*** | 0.792 | **.001*** |
| Olfactory_R | 0.092 | .078 | **0.028** | **<.001*** | 0.685 | **<.001*** |
| Frontal_Sup_Medial_L | 0.066 | .143 | -0.009 | .068 | **1.064** | **<.001*** |
| Frontal_Sup_Medial_R | 0.067 | .246 | *-0.013* | *.041^†^* | **1.222** | **<.001*** |
| Frontal_Med_Orb_L | 0.020 | .781 | **0.019** | **.015*** | 0.817 | **.001*** |
| Frontal_Med_Orb_R | 0.131 | .195 | **0.032** | **.007*** | 0.905 | **.006*** |
| Rectus_L | 0.150 | .037 | **0.022** | **.003*** | 0.831 | **<.001*** |
| Rectus_R | **0.190** | **.006*** | 0.012 | .099 | **0.633** | **.004*** |
| Insula_L | -0.021 | .661 | *0.013* | *.027^†^* | **1.145** | **<.001*** |
| Insula_R | 0.070 | .151 | **0.026** | **<.001*** | 0.801 | **<.001*** |
| Cingulum_Ant_L | -0.129 | .140 | **0.040** | **.001*** | 1.066 | **.001*** |
| Cingulum_Ant_R | -0.159 | .072 | **0.024** | **.018*** | 0.934 | **.002*** |
| Cingulum_Mid_L | -0.112 | .097 | 0.004 | .581 | **0.765** | **.001*** |
| Cingulum_Mid_R | **-0.193** | **.005*** | 0.005 | .469 | **0.631** | **.005*** |
| Cingulum_Post_L | -0.085 | .341 | -0.001 | .880 | **0.742** | **.007*** |
| Cingulum_Post_R | -0.119 | .221 | **-0.033** | **.004*** | **1.319** | **<.001*** |
| Hippocampus_L | 0.050 | .295 | -0.006 | .168 | **0.380** | **.009*** |
| Hippocampus_R | 0.023 | .627 | -0.014 | .010 | *0.255* | *.077* |
| ParaHippocampal_L | **0.104** | **.007*** | 0.007 | .089 | **0.826** | **<.001*** |
| ParaHippocampal_R | **0.118** | **<.001*** | **0.019** | **<.001*** | **0.674** | **<.001*** |
| Amygdala_L | 0.033 | .699 | -0.005 | .609 | **0.756** | **.009*** |
| Amygdala_R | 0.118 | .187 | **-0.042** | **.001*** | **1.223** | **<.001*** |
| Calcarine_L | 0.096 | .119 | **0.020** | **.011*** | **0.986** | **<.001*** |
| Calcarine_R | -0.016 | .815 | **0.043** | **<.001*** | **1.347** | **<.001*** |
| Cuneus_L | 0.078 | .087 | **-0.025** | **<.001*** | **1.231** | **<.001*** |
| Cuneus_R | 0.025 | .578 | -0.009 | .081 | **1.361** | **<.001*** |
| Lingual_L | 0.002 | .969 | 0.013 | .075 | **1.337** | **<.001*** |
| Lingual_R | 0.065 | .189 | **0.017** | **.006*** | **0.995** | **<.001*** |
| Occipital_Sup_L | -0.057 | .233 | -0.010 | .089 | **1.531** | **<.001*** |
| Occipital_Sup_R | 0.029 | .557 | -0.008 | .119 | **1.434** | **<.001*** |
| Occipital_Mid_L | -0.108 | .030 | -0.009 | .107 | **1.272** | **<.001*** |
| Occipital_Mid_R | 0.014 | .770 | -0.003 | .585 | **1.048** | **<.001*** |
| Occipital_Inf_L | -0.043 | .516 | **0.021** | **.014*** | **1.058** | **<.001*** |
| Occipital_Inf_R | 0.025 | .580 | 0.010 | .083 | **1.016** | **<.001*** |
| Fusiform_L | 0.042 | .274 | -0.008 | .092 | **1.131** | **<.001*** |
| Fusiform_R | 0.065 | .060 | -0.005 | .212 | **0.988** | **<.001*** |
| Postcentral_L | **-0.299** | **<.001*** | **0.028** | **.004*** | **1.202** | **<.001*** |
| Postcentral_R | -0.106 | .059 | **0.026** | **.001*** | **1.044** | **<.001*** |
| Parietal_Sup_L | -0.054 | .311 | -0.013 | .053 | **0.989** | **<.001*** |
| Parietal_Sup_R | 0.038 | .411 | -0.009 | .105 | **0.987** | **<.001*** |
| Parietal_Inf_L | **-0.183** | **.002*** | **-0.024** | **.002*** | **0.989** | **<.001*** |
| Parietal_Inf_R | -0.047 | .387 | **-0.027** | **.001** | **1.230** | **<.001*** |
| SupraMarginal_L | -0.007 | .917 | **-0.032** | **<.001*** | **1.012** | **<.001*** |
| SupraMarginal_R | -0.026 | .706 | **-0.036** | **<.001*** | **1.055** | **<.001*** |
| Angular_L | **-0.156** | **.005*** | **-0.015** | **.018*** | **1.008** | **<.001*** |
| Angular_R | -0.054 | .362 | -0.010 | .107 | **1.128** | **<.001*** |
| Precuneus_L | 0.007 | .823 | -0.007 | .079 | **0.909** | **<.001*** |
| Precuneus_R | 0.028 | .376 | *-0.009* | *.023^†^* | **1.027** | **<.001*** |
| Paracentral_Lobule_L | -0.091 | .117 | **0.032** | **<.001*** | **1.115** | **<.001*** |
| Paracentral_Lobule_R | -0.054 | .388 | **0.042** | **<.001*** | **0.840** | **<.001*** |
| Heschl_L | -0.037 | .742 | **0.039** | **.002*** | **1.473** | **<.001*** |
| Heschl_R | -0.015 | .875 | **0.058** | **<.001*** | **1.007** | **.001*** |
| Temporal_Sup_L | 0.024 | .591 | **-0.014** | **.017*** | **0.881** | **<.001*** |
| Temporal_Sup_R | 0.041 | .334 | -0.004 | .353 | **0.865** | **<.001*** |
| Temporal_Pole_Sup_L | **0.159** | **<.001*** | *0.011* | *.037^†^* | **0.775** | **<.001*** |
| Temporal_Pole_Sup_R | **0.159** | **.001*** | **0.018** | **.002*** | **0.598** | **<.001*** |
| Temporal_Mid_L | -0.014 | .784 | -0.010 | .095 | **0.860** | **<.001*** |
| Temporal_Mid_R | 0.018 | .686 | **-0.017** | **.003*** | **0.678** | **<.001*** |
| Temporal_Pole_Mid_L | 0.110 | .070 | 0.011 | .159 | **0.845** | **<.001*** |
| Temporal_Pole_Mid_R | 0.152 | .027 | *0.011* | *.046^†^* | **0.823** | **<.001*** |
| Temporal_Inf_L | -0.001 | .985 | -0.005 | .378 | **1.007** | **<.001*** |
| Temporal_Inf_R | 0.060 | .170 | -0.004 | .410 | **0.686** | **<.001*** |
| Unadjusted p-values are presented with those significant or trending following correction as noted:  *****Significant at p<.05 FDR corrected  *^†^*Trending at p<.10 FDR corrected | | | | | | |
